# Sex estimation through geometric morphometric analysis of the frontal bone: an assessment in pre-pubertal and post-pubertal modern Spanish population

**DOI:** 10.1007/s00414-021-02712-x

**Published:** 2021-10-25

**Authors:** Daniele Garcovich, Laura Albert Gasco, Alfonso Alvarado Lorenzo, Riccardo Aiuto, Milagros Adobes Martin

**Affiliations:** 1 Department of Dentistry, Universidad Europea de Valencia, Paseo de la Alameda, 7, 46010 Valencia, Spain; 2 Department of Dentistry, Universidad de Salamanca, Salamanca, Spain; 3 Department of Oral Rehabilitation, Instituto Stomatologico Italiano, Universitá Di Milano, Milan, Italy

**Keywords:** Sex estimation, Sexual dimorphism, Pre-pubertal, Post-pubertal, Frontal bone, Forensic anthropology

## Abstract

Sex estimates is a key step of biological profile assessment in a forensic or anthropologic context. In this study, the sexual dimorphism of the frontal bone was analyzed to assess the accuracy of sex estimates using a geometric morphometric approach in a pre-pubertal and post-pubertal sample. The shape of the frontal bone was digitized on the lateral cephalograms of 87 pre-pubertal subjects (42 males, mean age 10.14, SD ± 1.48 years; 45 females mean age 10.02, SD ± 1.11 years) and 103 post-pubertal ones (53 males, mean age 29.33 SD ± 11.88 years; 50 females, mean age 26.77 SD ± 11.07 years). A generalized Procrustes analysis (GPA) was performed for shape analyses, filtering the effects of position, rotation, translation, and size. A principal component analysis (PCA) was performed on the GPA transformed variables, and a multiple logistic regression model was used to assess the accuracy of sex estimates. In both age groups, the average size of the centroid was significantly larger in males. The females presented shapes with a shorter distance between P2 (glabella) and P1 (supratoral) and a general narrowing of the structure on the sagittal plane. In the pre-pubertal group, the shape difference was not statistically significant. In the post-pubertal group, the mean shape was significantly different between the sexes. The method displayed a high accuracy for sex estimates (88.7% males, 90.3% females) also when applied in a validation sample (82.6% males and 94.1% females). The described morphometric analysis of the frontal bone is based on a limited number of landmarks, which allows sex estimates with high accuracy in post-pubertal subjects, while it is not applicable in pre-pubertal ones.

## Introduction

In a forensic context, sex estimates is a key step in the biologic profile assessment of unknown human remains. The most reliable bone for sex estimates is os coxae, whose sexual dimorphism is not population-specific and showed a classification accuracy higher than that of the entire pelvis [1]. The pelvic assessment is followed in accuracy by postcranial metrics, and then morphology and metrics of the skull [2]. In some instances, the most reliable skeletal regions may be missing or too badly damaged for analyses. The cranium, more resistant to taphonomic phenomena, can be used for this purpose or to corroborate estimates based on the pelvis [3–5]. Many cranial areas proved to be extremely valuable for sex estimates, such as the mastoid process, the mental region of the mandibula, the superior margin of the orbital rim, and the frontal or glabellar area [6].

Sex estimates can be performed through a morphological examination of traits significantly associated with sex. The accuracy of this type of assessment depends on the operator’s training and experience, which is subjective and characterized by a low intra- and inter-operator reliability due to the qualitative nature of the data [7].

Dimensional analyses based on linear measurements, commonly used in anthropometric protocols, are objective and demonstrate a certain accuracy but rely only on size and can poorly discriminate the sex of an individual when norms gathered from a population with larger cranial dimensions are applied to a population with smaller dimensions [8].

Geometric morphometry is an effective and validated method of describing and analyzing the geometry and configuration of a set of landmarks. They enable discrimination between shape and size and eliminate the shape variables that depend on size from those that are functions of other variables, as is the case for the sex of an individual [9]. A geometric morphometric analysis enables to draw suggestive diagrams of morphological transformations or differences, allowing easy visualization of the shape and its variations. These diagrams are easy to understand and allow for a straightforward way of data interpretation and communication [10].

Sex estimation methods are generally not reliable in subadult groups because dimorphic sexual traits are not fully developed, while shape and size differences become more significant as age increases [11]. Lateral cephalometric measurements showed promising accuracy in population-specific samples of subadults, ranging from the 78 to 89% reported by Gonzalez to the 95% reported by Hsiao [12, 13].

To our best knowledge, the sexual dimorphism of the frontal bone on lateral cephalometric radiographs was studied only once before using a geometric morphometric approach [14]. The assessment was carried out in a Latin American sample of 60 adult individuals. The aim of our study was to evaluate the sexual dimorphism of the frontal bone in a larger sample of individuals of Spanish ancestry. The analysis was performed in both a pre-pubertal and a post-pubertal cohort to assess its variation with age.

## Materials and methods

### Reference sample

The sample size of the study was previously calculated using R 3.5.1 software (R Core Team, 2013. R: a language and environment for statistical computing. R Foundation for Statistical Computing, Wien, Austria). It was determined that at least 80 subjects were required to achieve a power of 90% for a *δ* value of 1.0 (*α* = 0.05).

The main sample consisted of 190 lateral cephalometric radiographs performed on contemporary Spanish individuals seeking orthodontic treatment in the Department of Orthodontics of the Universidad Europea de Valencia. All images were captured from January 2017 to May 2020. Sex and age and other demographic data were recorded at the time of lateral cephalometric radiograph acquisition.

The sample was selected according to the following inclusion criteria: radiographs of excellent quality and contrast; males between the ages of 7 and 12.5 years; males older than 14 years; females between the ages of 7 and 10 years; females older than 13.4 years; Spanish ancestors from at least two previous generations; CVS (cervical vertebrae maturation stages) 1 and 2 for individuals in the pre-pubertal group; CVS 5 and 6 for individuals in the post-pubertal group.

Exclusion criteria: presence of craniofacial syndromes; history of craniofacial trauma; previous orthodontic or orthognathic treatment; history of onset of menarche or voice break for individuals within the pre-pubertal group; CVS 3 to 6 for individuals in the pre-pubertal group; CVS 1 to 4 for individuals in the post-pubertal group.

The final sample was divided into two sub-samples of 87 radiographs from subjects in the pre-pubertal stage (42 males, mean age 10.14, SD ± 1.48 years; 45 females, mean age 10.02, SD ± 1.11 years) and 103 radiographs from individuals in the post-pubertal stage (53 males, mean age 29.33, SD ± 11.88 years; 50 females, mean age 26.77, SD ± 11.07 years).

### Validation sample

To validate the results of the method in the post-pubertal sample, a validation sample of 40 lateral cephalometric radiographs was selected according to the same inclusion and exclusion criteria among those performed from May 2020 to July 2021 in the same university department. The main sample and the validation one can thus be considered belonging to the same population group. The validation sample consisted of 40 individuals (20 males, mean age 32.45, SD ± 10.2 years; 20 females, mean age 31.9, SD ± 9.11 years).

### Radiographic technique and landmark positioning

All radiographs were taken with the Frankfort horizontal parallel to the ground and the patient positioned in a cephalostat, using a Kodak 9003D digital panoramic system under the same parameters (exposure setting, 80 kV and 10 mA; exposition time, 0.04 s; magnification 1:1). The records were stored in jpg format.

**Fig. 1:**
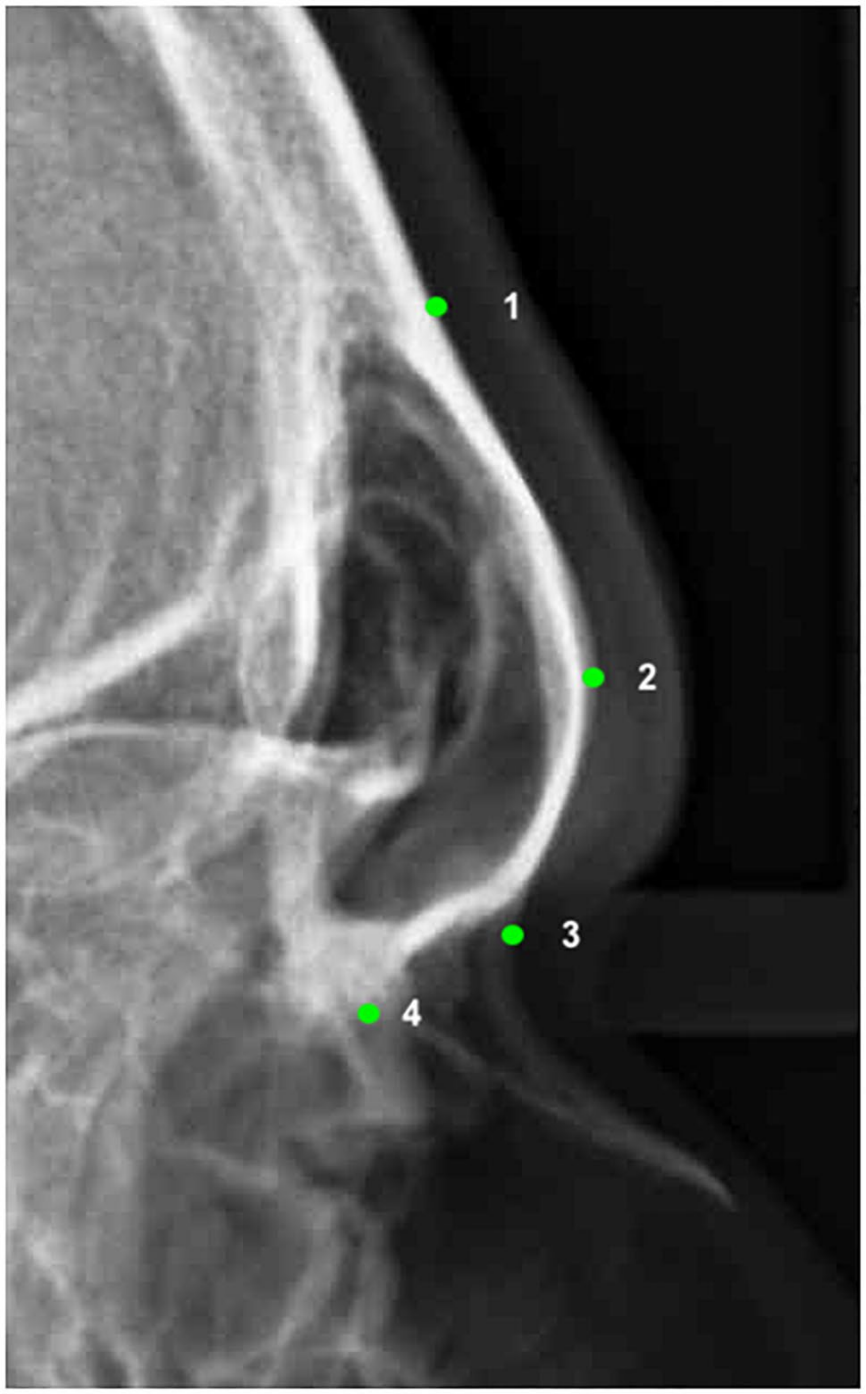
The landmarks used in the geometric morphometric analysis as traced on a lateral cephalogram

Four landmarks were digitized on the selected radiographs using cephalometric software (NemoCeph®, Nemotec, Madrid, Spain), two in the squamous part (landmarks 1 and 2) and two in the nasal part (landmarks 3 and 4) of the frontal bone (Fig. 1, Table 1). Landmark 1 can be classified as type III according to the Bookstein classification (extremal points), landmark 2 is a type II landmark (maxima of curvature points), and landmarks 3 and 4 are type I landmarks (discrete juxtapositions of tissues) being sutures intersections.

**Table 1:** Definition of the landmarks digitized in the frontal bone. The type of landmark according to the Bookstein classification is indicated

Landmark	*N*	Definition	Type
Supratoral sulcus	1	Point on the outer surface of the frontal bone where starts the protrusion of the glabellar zone	III
Glabella	2	The most prominent point at the midline of the squamous part of the frontal bone	II
Nasion	3	The most anterior point on the frontonasal suture	I
B point	4	Point where the frontomaxillary meets the nasomaxillary suture	I

To assess intra-operator reliability, a group of 15 randomly selected radiographs belonging to the study group were traced twice at a 2-week interval by the principal investigator (LA). The inter-operator reliability was assessed by retracing the same radiographs by a second operator (DG). To ensure a single-blind assessment of the records, another member of the research group (MA) selected and coded the sample according to the inclusion criteria so that the operators were unaware of the age and sex of the records.

### Statistical analysis

The method error, the degree of reproducibility, and the intra- and inter-operator reliability were estimated by the intra-class correlation coefficient (ICC), by the technical error of measurement (TEM), and by the relative technical error of measurement (rTEM). A confidence interval of 95% (95% CI) was used. A Welch *t*-test for independent samples was employed to test the hypothesis of homogeneity between the centroid mean size of the male and female samples. A generalized Procrustes analysis (GPA) was performed to achieve optimal superimposition of the landmark configuration minimizing the sum of the squared deviations between the landmarks after filtering the effects of position rotation, translation, and size. A Goodall *F* test for independent samples was used to test the hypothesis of similar average size in both sexes. A principal component analysis (PCA) was performed on the GPA transformed variables. The components are variable in shape and allow one to understand the relative change in shape along the respective range. A thin-plate spline (TPS) function was used to visually represent the differences in shape between the two groups. Finally, a multiple logistic regression model was used to assess the accuracy of sex estimates using the factor scores of principal components. Odds ratios and 95% confidence intervals were given. The significance level was set at 5% (*α* = 0.05).

Statistical analysis was performed using R 3.5.1 software (R Core Team 2013) and SPSS software (version 15.0; IBM Corp., Armonk, NY, USA).

## Results

### Method error

The ICC estimated from repeated measurements had a value greater than 0.95. The TEM ranged from 0 to 0.05 mm, while the rTEM ranged from 0 to 2.15%. All values indicated a low method error and a high intra-operator reliability. On the other hand, the ICC regarding inter-operator reliability was greater than 0.90, the TEM ranged from 0 to 0.2 mm, while the relative rTEM ranged from 0 to 3.45% suggesting high repeatability of the measurements. For both intra- and inter-operator reliability, the highest values of TEM and rTEM were displayed by landmarks 1 and 2.

### Pre-pubertal group

**Fig. 2:**
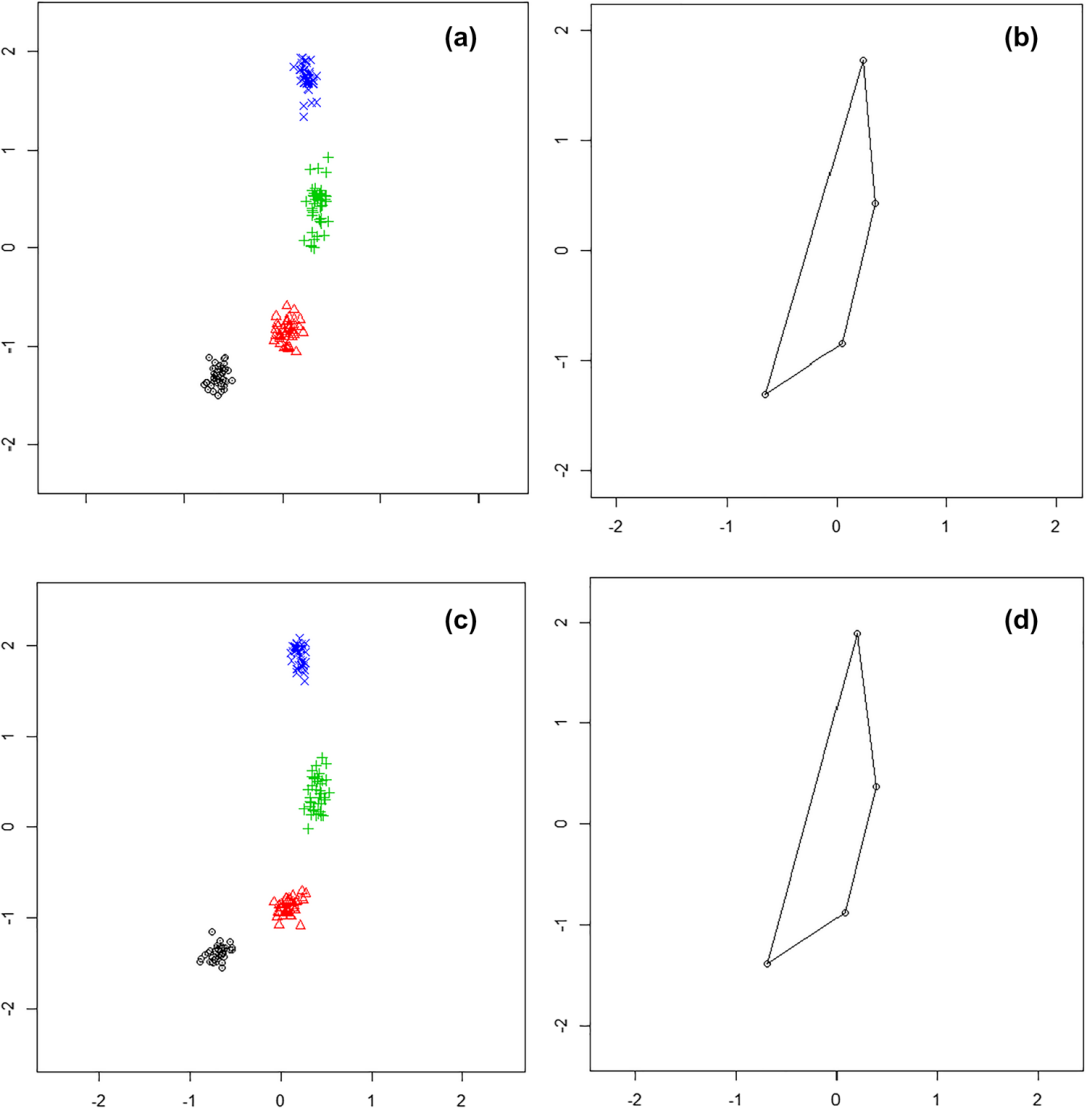
Position of the landmarks after applying the GPA to the female (**a**) and male (**c**) groups in the pre-pubertal group. The mean shape in the female (**b**) and male (**d**) groups in the same age cohort

In male individuals, the mean size of the centroid is 2.65 ± 0.34 mm and the shape variability shows a root mean square deviation (RMSD) of 0.104. In the female group, the mean size of the centroid is 2.49 ± 0.26 mm, while the shape variability is higher than in the male group, being the RMSD equal to 0.123. The mean difference between the two groups is 0.19 mm (95% CI: 0.03–0.29). When the mean size of the centroid is compared between the two groups using a Welch *t*-test, a significant difference is found (*t* = 2.42, *p* = 0.018, *p < 0.05), but when the shapes are compared, after GPA application, using a Goodall *F* test, a non-significant difference is highlighted (*F* = 2.94, *p* = 0.054, *p < 0.05) (Fig. 2).

**Fig. 3:**
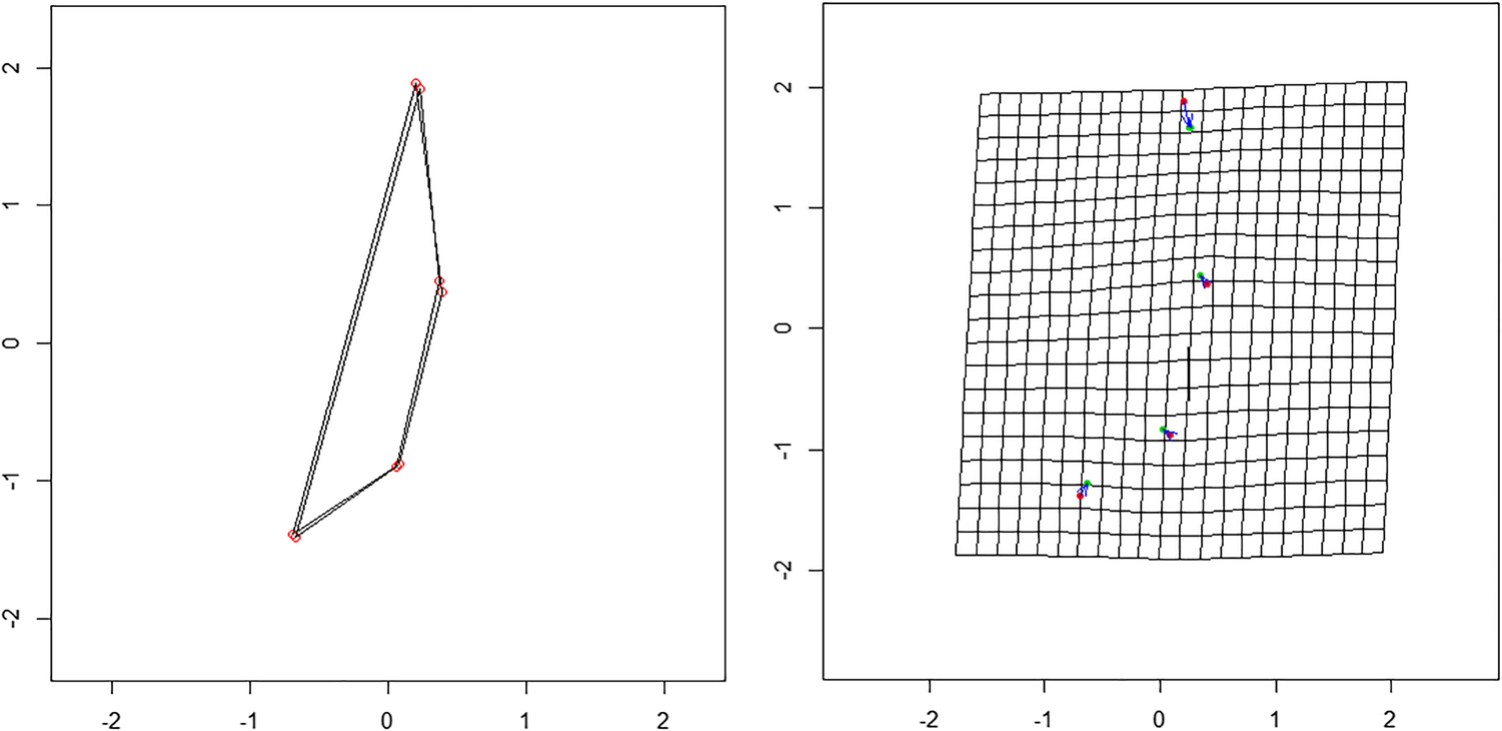
Superimposition of the mean shape in the pre-pubertal male and female groups (**a**). Representation on a TPS grid of the deformation needed to change the shape; vectors have a male to female direction

When an overall Procrustes analysis (OPA) is applied to the two groups, an optimal superimposition of the shapes is obtained. The shapes are clearly similar, and in the thin-plate spline grid, the different vectors show the relative direction of the difference between one configuration and another (Fig. 3). Due to the small difference in shape, the vectors’ magnitude is low. The PCA applied to the GPA data highlights how out of the eight principal components (PC), the first three explain 94.5% of the variance (PC1 = 67.1%; PC2 = 15.8%; PC3 = 11.6%).

**Fig. 4:**
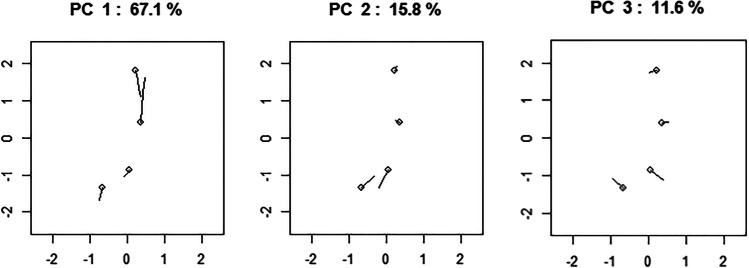
The direction of variation of PC1, PC2, and PC3 in the pre-pubertal group. The round marker represents the mean position of a landmark in the group. The percentage of variance explained by each component is indicated

The directions of variation of the first three PCs are reported in Fig. 4. PC1 is the vertical distance between points 2 (glabella) and 1 (supratoral sulcus); PC2 is the proximity between points 3 (point B) and 4 (nasion); PC3 represents how points 1 and 4 move away from points 2 and 3 in the sagittal plane.

Applying a binary logistic regression model, it is possible to assess how the scores of PC1, PC2, and PC3 are not significantly related to the sex of an individual (Table 2). The logistic model can be expressed by the following equation, in which *p* is the probability of being female:

**Table 2:** Odds ratio (OR) and 95% confidence interval (CI) for sex of an individual, in the pre-pubertal sample, relative to the score of PC1, PC2, and PC3. *p < 0.05; **p < 0.01; ***p < 0.001

	95% CI			
	OR	Lower	Upper	***p***
PC1	5.72	0.90	36.2	0.064
PC2	15.0	0.35	640.9	0.157
PC3	0.06	0.00	4.96	0.213
(Constant)	1.08			0.731

$$\frac p{1-p}=1.08\;{5.72}^{\mathrm{PC}1}{\;15.04}^{\mathrm{PC}2}{\;0.06}^{\mathrm{PC}3}$$

The discriminating accuracy of the model can be considered low (57.1% males and 62.2% females).

### Post-pubertal group

In male individuals, the mean size of the centroid is 2.85 ± 0.31 mm and the shape variability displays an RMSD of 0.116. In the female group, the mean size of the centroid is 2.59 ± 0.23 mm, while the shape variability is lower than in the male group, RMSD is equal to 0.103.

**Fig. 5:**
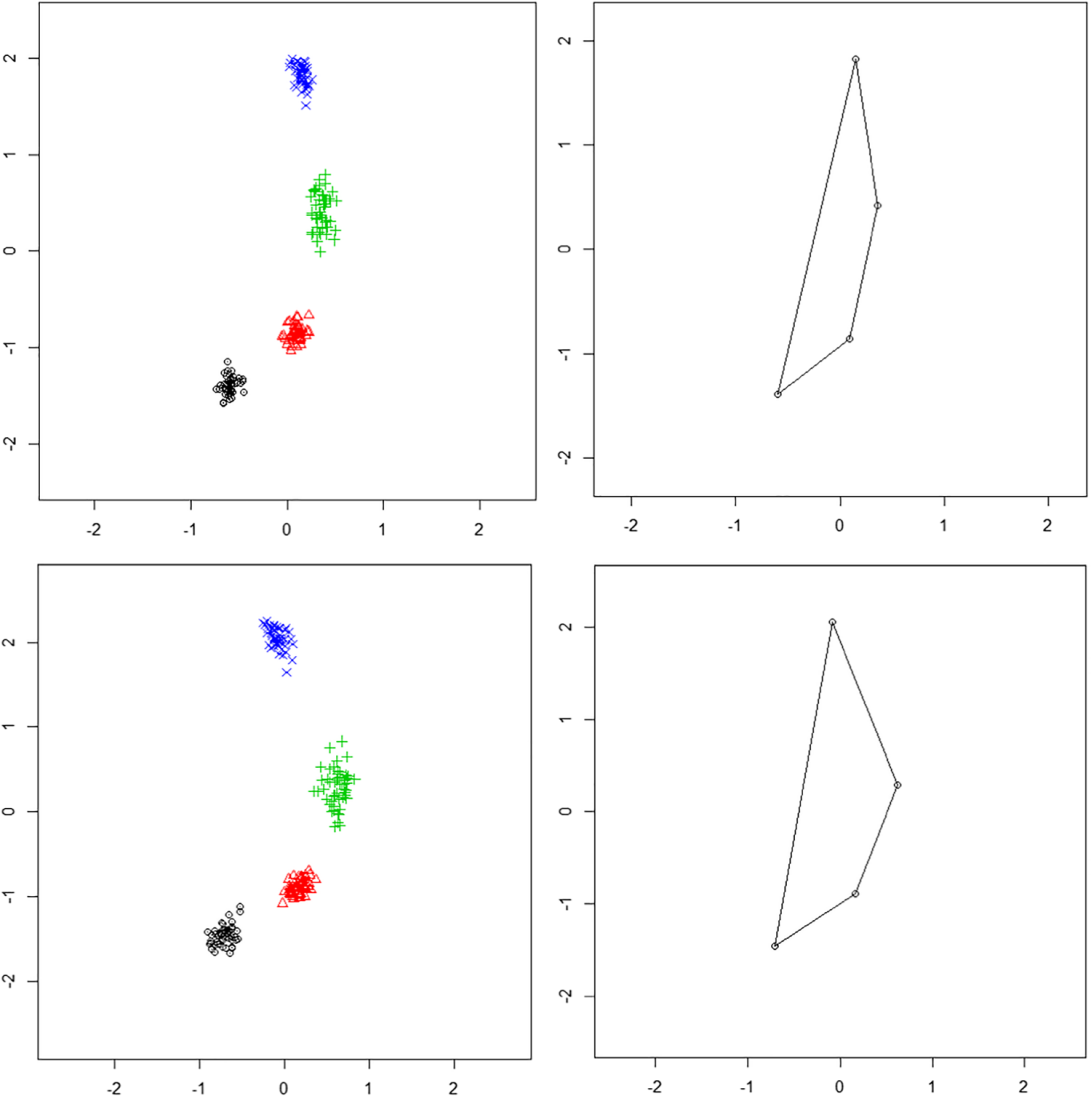
Position of the landmarks after applying the GPA to the (**a**) female and (**c**) male groups in the post-pubertal group. The mean shape in the female (**b**) and male (**d**) groups in the same age cohort

The mean difference between the two groups is 0.26 mm (95% CI: 0.16–0.37). When the mean size of the centroid is compared between the two groups using a Welch *t*-test, a significant difference is found (*t* = 4.87, *p* < 0.001***). When the shapes are compared after GPA application, using a Goodall *F* test, a highly significant difference is highlighted (*F* = 15.61, *p* < 0.001***) (Fig. 5).

**Fig. 6:**
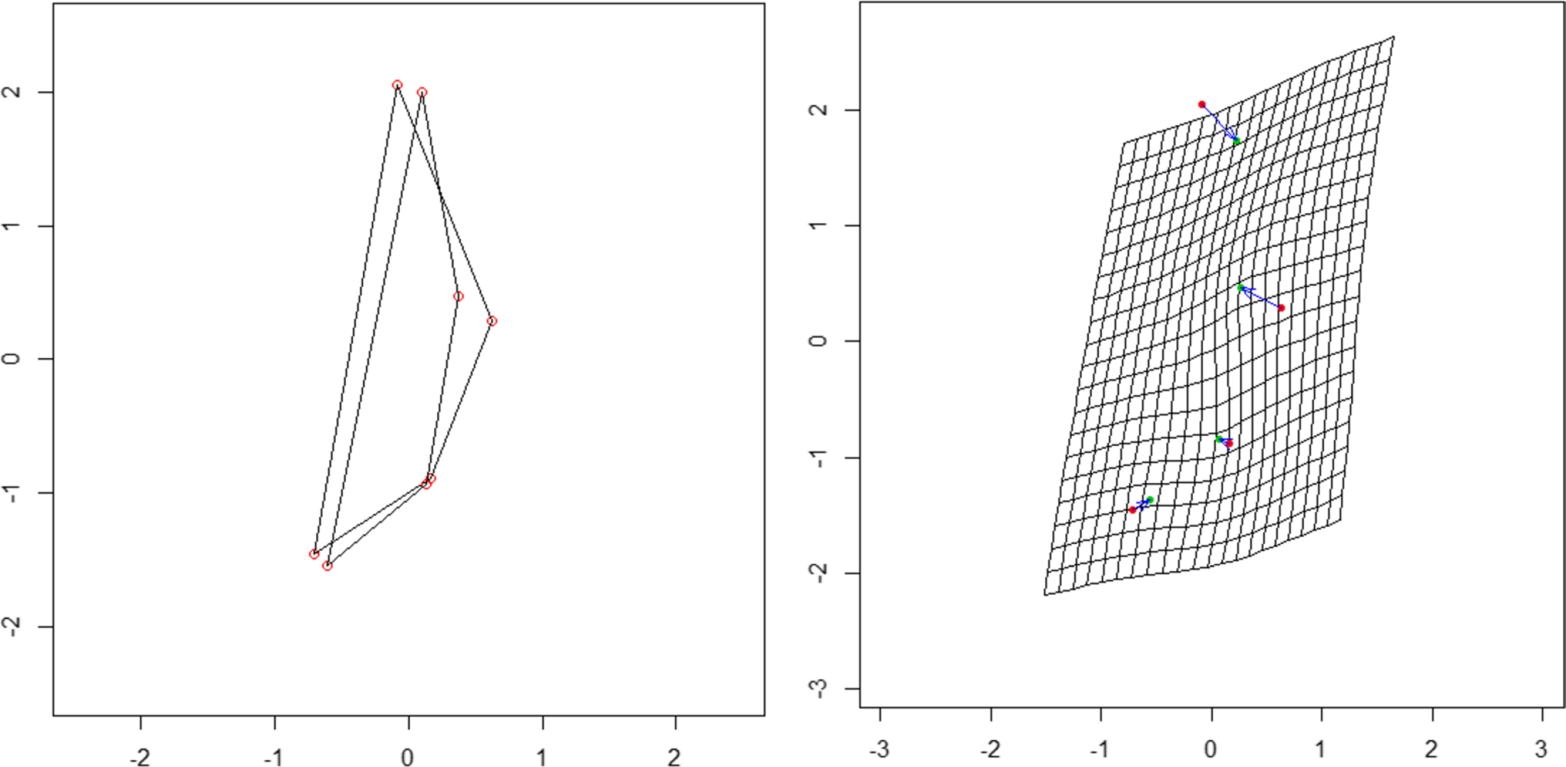
Superimposition of the mean shape in the post-pubertal male and female group (**a**). Representation on a TPS grid of the deformation needed to change the shape; vectors have a male to female direction

When an OPA is applied to the two groups, an optimal superimposition of the shapes is obtained. In the thin-plate spline grid, the different vectors show the relative direction and magnitude of the difference between one configuration and another; the magnitude of the change is marked at landmarks 1 and 2 (Fig. 6).

**Fig. 7:**
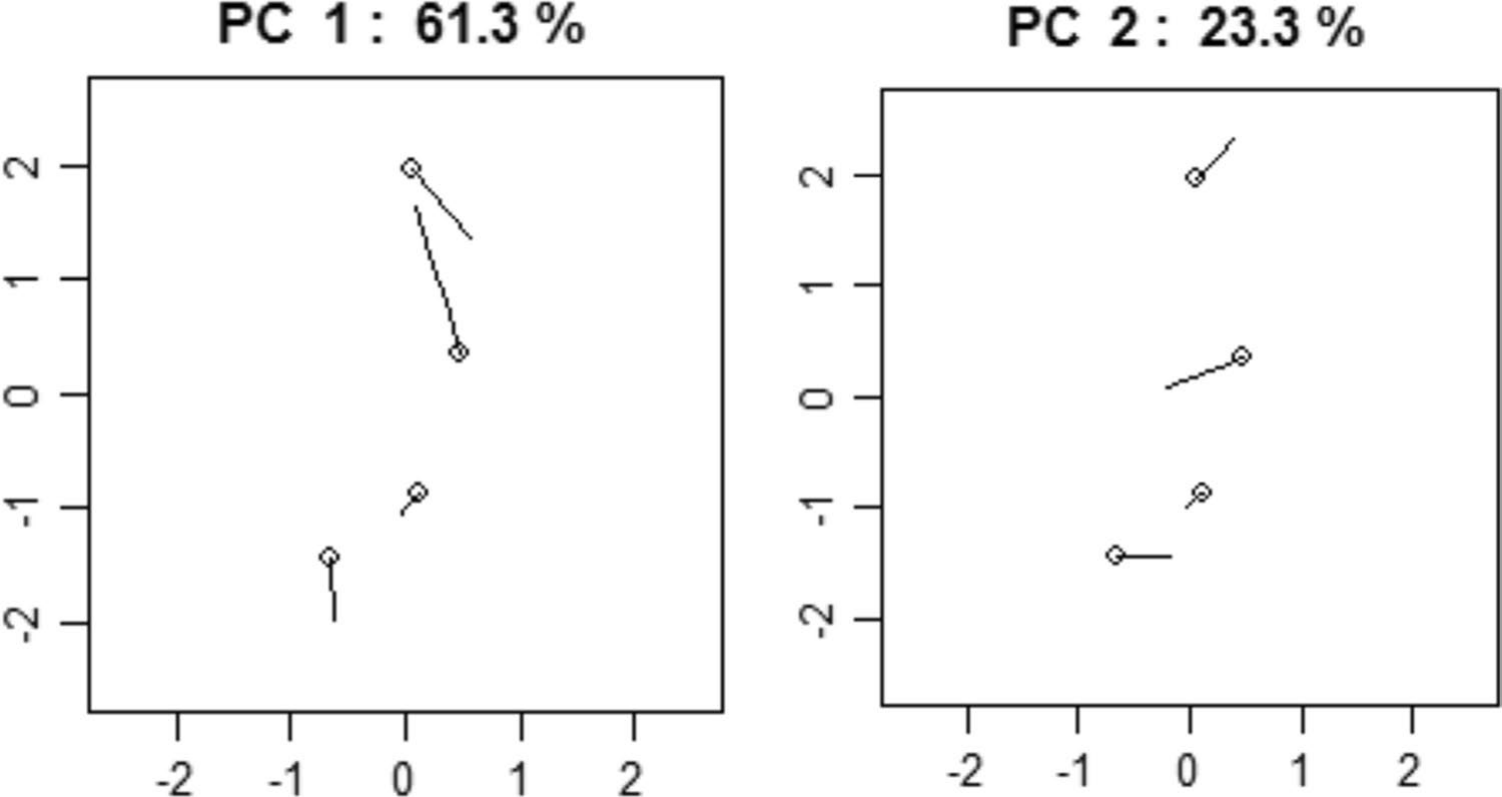
The direction of variation of PC1 and PC2 in the post-pubertal group. The round marker represents the mean position of a landmark in the group. The percentage of variance explained by each component is indicated

The PCA applied to the GPA data highlights how out of the eight PCs the first three explain 94.6% of the variance (PC1 = 61.3%; PC2 = 23.4%; PC3 = 9.93%). The directions of variation of the first two PCs are reported in Fig. 7. PC1 is the vertical distance between landmarks 2 (glabella) and 1 (supratoral sulcus); PC2 represents landmarks 1 and 3 move closer to points 2 and 4, and this mutual displacement reflects a narrowing of the shape; PC3 represents how landmarks 3 (point B) and 4 (nasion) get closer, narrowing, and shortening the shape.

Applying a binary logistic regression model, it is possible to assess how the scores of PC1 and PC2 are significantly related to the sex of an individual (Table 3). The logistic model can be expressed by the following equation in which *p* is the probability of being female:

**Table 3:** Odds ratio (OR) and 95% confidence interval (CI) for sex of an individual, in the post-pubertal sample, relative to the score of PC1, PC2, and PC3. *p < 0.05; **p < 0.01; ***p < 0.001

	95% CI			
	OR	Lower	Upper	***p***
PC1	6454.8	147.9	281,672	< 0.001***
PC2	1.07 × 10^7^	10,950	1.04 × 10^11^	< 0.001***
PC3	0.39	0.00	328.5	0.782
(Constant)	0.70			0.367

Note: 0.70 and 6454.8 should be multiplied are two different factors

$$\frac p{1-p}=0.70\;{6454.8}^{\mathrm{PC}1}{(1.07\mathrm x10^7)}^{\mathrm{PC}2}\;{0.39}^{\mathrm{PC}3}$$

The discriminating accuracy of the model can be considered high (88.7% males and 90.3% females).

The discriminating accuracy of the method when applied in the validation sample was only slightly lower in the male sub-group than the one obtained in the main sample (82.6% males and 94.1% females).

## Discussion

To our best knowledge, this is the first study addressing frontal bone sexual dimorphism in a pre-pubertal and post-pubertal pool of Spanish individuals through a geometric morphometric approach performed on lateral cephalometric radiographs.

DNA analysis is the gold standard for sex estimates in a forensic setting, but in some contexts, a morphological approach is preferred because it is more cost-effective (i.e., genocide or mass disasters) or for being the only viable one due to a too small, altered, or degraded sample, as is the case in a paleoanthropological context [15].

The forehead and frontal bone anatomy are sexually dimorphic and important in determining an individual facial appearance to such an extent that facial feminization surgery focused on reshaping the forehead at first. The increase in frontal sinus thickness and supraorbital ridges, as well as a flatter forehead, are classically associated with male facial appearance [16].

Several authors investigated the sexual dimorphism of the frontal bone. Williams et al. (2006) in an osteological sample of 50 white individuals of European descent found a high accuracy when the morphological evaluation was performed using the size of the supraorbital ridge (88%), while in his sample the frontal eminence did not obtain the same accuracy (64%) [17].

Nikita E. (2019) in a quantitative assessment of cranial traits on photographic imaging highlighted how discriminant variables relative to frontal bone were those displaying the most accurate classification rate (84.2 to 87.3%) [18].

Del Bove et al. (2019) using a morphometric approach on the outer surface of the frontal bone established a hierarchy of importance of sexually dimorphic areas of the frontal bone. Their study highlighted how the supraorbital ridges and the glabellar region were the most valuable areas for sex estimation, while the variable size, of frontal bone measurements, was not strongly related to sex [19].

Shearer et al. (2012) focused on the browridge volume as a quantitative discriminant for sex estimates and found an accuracy of 75%, with a marked variability according to the ethnicity of the sub-sample. Garvin and Ruf (2012) using a surface laser scanner could correctly estimate the sex in 91.6% of the individuals using a morphometric assessment of the skeletal browridge on dry skulls [20].

Garvin et al. (2014) pointed out that despite the good accuracy of the glabellar region for sex estimates, all cranial traits are population-specific, highlighting the need to adapt qualitative or quantitative assessments to ethnicity. The strong effect of ethnicity on method accuracy was also underlined by Petaros et al. (2017). According to their results, the frontal inclination angles of the glabella and supraglabella, measured on 3D models, could estimate sex with an accuracy rate ranging from 75 to 81% in US White, US Black, and Portuguese groups, while the accuracy decreased to 66% in the Chinese group.

Perlaza et al. (2014), through morphometric analyses based on the same landmarks used in the present study, obtained an accuracy of 84.31%. Consistent with our results, also in his sample, PC1 (58.8%) and PC2 (23.34%) accounted for the vast majority of the variance, with the narrowing of the shape in the sagittal plane being the most differential feature of the female sex [14]. This author, as in our study, used a pool of lateral cephalograms selected from a modern Latin American sample and not an osteological sample as in the vast majority of the published research [14].

Gonzalez et al. (2012), applying a geometric morphometric approach to 125 dry skulls, obtained a lower accuracy of 77.86% and, according to what was reported by other authors, questioned the applicability of discriminant functions derived from one sample to others, calling for population-specific standards to improve sexing accuracy [21]. In their analyses, the outline of the frontal bone, as well as the zygomatic arch and the mastoid, was included. Koelzer et al. (2019) also focused on the outline of the frontal bone from a sagittal point of view. Using a set of 211 postmortem computed tomography (CT) scans, they related the slope or inclination of the forehead, measured as the inclination of the frontal outline with Frankfort horizontal, to the sex of an individual with an overall accuracy of 80% [5]. Bulut et al. (2016) reported a 77.5% accuracy when the differences in the roundness of the external shape of the frontal bone between the sexes were assessed on CT scans using a landmark-free method [7].

Despite the accuracy displayed by the described method in our population, the evidence from studies comparing the accuracy of sex estimation through traditional biological anthropology approaches (metric and morphological) to known sex from DNA analyses highlighted how the precision increases as more of the skeletal remains are available for analysis [22]. In a forensic context, sex estimates based on a single indicator, as in this case, the frontal bone morphology, should, therefore, be avoided. The inaccuracy of the sex estimate can be dramatically reduced by using a multimethod approach [2].

One of the strengths of our approach is the limited number of landmarks needed and the relatively small area used for the assessment, which makes it also viable in the case of a fragmented sample. In these cases, traditional linear measurement methods can be difficult to apply, and sexing accuracy decreases while a geometric morphometric approach can still obtain a good accuracy without analyzing the whole cranium. The method based on digital radiographs can also be employed in the cases of semi-fleshed, charred, or otherwise highly decomposed and degraded samples where maceration cannot be tried prior to the analysis [23].

Moreover, the landmarks are easy to locate since they are clearly defined and related to midline structures, less affected by the distortion inherent to the radiologic technique [24]. The morphometric approach used in the study adds to the discriminating value of the outer profile of the frontal bone as defined by the landmarks on the squamous region, the sagittal dimension defined by the landmarks on the frontonasal region, which are affected by sinus pneumatization and enlargement during puberty. Our findings are supported by those of Čechová et al. (2019), who reported accuracy of 83.49% when a morphometric approach was applied to the external surface of the frontal bone, increasing to 98.05% when the frontal sinus was assessed simultaneously [25].

The accuracy of the described morphometric method is high (88.7–90.3%) and unlike the methods relying on computed tomography (CT) and magnetic resonance imaging (MRI), the 2D imaging system used is easily available, cost-efficient, and does not require a complex operator training. The cost-efficiency of the procedure is especially important when a large number of individuals should be assessed. The sophisticated statistical approach, a common feature of all geometric morphometric approaches, makes it a time-consuming procedure and could discourage its use as a routine procedure. At a global level, the method can be a useful component in a multifeature or multimodal system for statistical sex estimation.

To select the pre-pubertal and post-pubertal groups, we considered the recently published, large cohort studies in the north European population. In the male group, the voice break considered a pubertal milestone appears at a mean age of 13 to 14 years depending on the author. We decided to include only individuals older than 14 years and leave out the records of males between the age of 12.5 and 14 years of age where the onset of puberty was not guaranteed [26–29]. The age of inclusion in the female group was set on the basis of the age of menarche onset, as reported by various authors in contemporary populations, which was between 12.5 and 13.4 years. Females between 10.5 and 13.4 years were excluded [27, 28, 30]. To ensure a correct allocation of the individuals in each group, we assessed the radiographs according to the cervical vertebrae maturation stage as described by Baccetti et al. (2000) [31]. The evaluation can be done on the same lateral radiograph used for the geometric morphometric evaluation and has proven to be as reliable as the hand-wrist radiograph in predicting the pubertal growth spurt [32, 33].

In the pre-pubertal sample, significantly different size of the centroid was found, but the shape was not different between sexes. The logistic regression displayed how neither of the principal components were related to the sex of an individual, and the method proved to be not applicable in this age group. The high dimorphism displayed by the frontal bone in the post-pubertal cohort partly explains the low accuracy of the method in the pre-pubertal one.

The low accuracy of sex estimates in subadults is a major problem in the biologic profile assessment of an entire population. Even the most reliable bone of the human skeleton in adult individuals shows low reliability in pre-pubertal subjects, Estevez Campos et al. in 2018 highlighted the low sexual dimorphism of the ischium and pubis [11]. Other areas such as the ilium or the mandible have shown promising results, but they could not be confirmed in further validation studies [34–36].

## Conclusions

A geometric morphometric assessment of the frontal bone in a contemporary Spanish population was assessed in post-pubertal and pre-pubertal age groups. The method presented remarkable reliability with a low intra- and inter-observer error. Through the tracing of a limited number of landmarks allowed for sex estimates with a high discriminating accuracy (88.7% male and 90.3% female) in the post-pubertal group. The size of the centroid is significantly different in both sexes. Due to the limited area explored, the method could be feasible also in case of fragmented and not well-preserved remains. In the pre-pubertal group, despite the significant difference in size between the centroids of the two groups, the method presented a low discriminating accuracy (57.1% males and 62.2% females). Shapes should be considered similar, and the method is not valid for sex discrimination.

## Data Availability

All relevant datasets used and/or analyzed during the current study are within the paper; any additional dataset is available from the corresponding author on reasonable request.

## References

[1] Kranioti EF, Šťovíčková L, Karell MA, Brůžek J (2019). Sex estimation of os coxae using DSP2 software: a validation study of a Greek sample. Forensic Sci Int.

[2] Klales A (2020). Sex estimation of the human skeleton, 1st editio.

[3] Zaafrane M, Ben Khelil M, Naccache I (2018). Sex determination of a Tunisian population by CT scan analysis of the skull. Int J Legal Med.

[4] Goyal M, Acharya AB, Sattur AP, Naikmasur VG (2013). Are frontal sinuses useful indicators of sex?. J Forensic Leg Med.

[5] Koelzer SC, Kuemmel IV, Koelzer JT (2019). Definitions of frontal bone inclination: applicability and quantification. Forensic Sci Int.

[6] Daniele G, Matilde S-SA, María M (2020). Sex estimation by tooth dimension in a contemporary Spanish population. Forensic Sci Int.

[7] Bulut O, Petaros A, Hizliol I (2016). Sexual dimorphism in frontal bone roundness quantified by a novel 3D-based and landmark-free method. Forensic Sci Int.

[8] Kimmerle EH, Ross A, Slice D (2008). Sexual dimorphism in America: geometric morphometric analysis of the craniofacial region. J Forensic Sci.

[9] Rosas A, Bastir M (2002). Thin-plate spline analysis of allometry and sexual dimorphism in the human craniofacial complex. Am J Phys Anthropol.

[10] Webster M, Sheets HD (2010). A practical introduction to landmark-based geometric morphometrics. Paleontol Soc Pap.

[11] Estévez Campo EJ, López-Lázaro S, López-Morago Rodríguez C (2018). Specific-age group sex estimation of infants through geometric morphometrics analysis of pubis and ischium. Forensic Sci Int.

[12] Hsiao TH, Tsai SM, Chou ST (2010). Sex determination using discriminant function analysis in children and adolescents: a lateral cephalometric study. Int J Legal Med.

[13] Gonzalez RA (2012). Determination of sex from juvenile crania by means of discriminant function analysis*,†. J Forensic Sci.

[14] Perlaza NA (2014). Sex determination from the frontal bone: a geometric morphometric study. J Forensic Sci.

[15] Quincey D, Carle G, Alunni V, Quatrehomme G (2013). Difficulties of sex determination from forensic bone degraded DNA: a comparison of three methods. Sci Justice.

[16] Morrison SD, Vyas KS, Motakef S, et al (2016) Facial feminization: systematic review of the literature. Plast. Reconstr. Surg. 10.1097/PRS.0000000000002171 27219232

[17] Williams BA, Rogers TL (2006). Evaluating the accuracy and precision of cranial morphological traits for sex determination. J Forensic Sci.

[18] Nikita E (2019). Quantitative sex estimation based on cranial traits using R functions. J Forensic Sci.

[19] Del Bove A, Profico A, Riga A (2020). A geometric morphometric approach to the study of sexual dimorphism in the modern human frontal bone. Am J Phys Anthropol.

[20] Garvin HM, Ruff CB (2012). Sexual dimorphism in skeletal browridge and chin morphologies determined using a new quantitative method. Am J Phys Anthropol.

[21] Franklin D, Freedman L, Milne N (2005). Sexual dimorphism and discriminant function sexing in indigenous South African crania. HOMO- J Comp Hum Biol.

[22] Thomas RM, Parks CL, Richard AH (2016). Accuracy rates of sex estimation by forensic anthropologists through comparison with DNA typing results in forensic casework. J Forensic Sci.

[23] KrishanChatterjeeKanchan KPMT (2016). A review of sex estimation techniques during examination of skeletal remains in forensic anthropology casework. Forensic Sci Int.

[24] Sayinsu K, Isik F, Trakyali G, Arun T (2007). An evaluation of the errors in cephalometric measurements on scanned cephalometric images and conventional tracings. Eur J Orthod.

[25] Čechová M, Dupej J, Brůžek J (2019). Sex estimation using external morphology of the frontal bone and frontal sinuses in a contemporary Czech population. Int J Legal Med.

[26] Juul A, Magnusdottir S, Scheike T (2007). Age at voice break in Danish boys: effects of pre-pubertal body mass index and secular trend. Int J Androl.

[27] Juul A, Teilmann G, Scheike T, et al (2006) Pubertal development in Danish children: comparison of recent European and US data. In: International Journal of Andrology 10.1111/j.1365-2605.2005.00556.x 16466546

[28] Brix N, Ernst A, Lauridsen LLB (2019). Timing of puberty in boys and girls: a population-based study. Paediatr Perinat Epidemiol.

[29] Busch AS, Hollis B, Day FR (2019). Voice break in boys-temporal relations with other pubertal milestones and likely causal effects of BMI. Hum Reprod.

[30] Biro FM, Pajak A, Wolff MS (2018). Age of menarche in a longitudinal US cohort. J Pediatr Adolesc Gynecol.

[31] Franchi L, Baccetti T, McNamara JA (2000). Mandibular growth as related to cervical vertebral maturation and body height. Am J Orthod Dentofac Orthop.

[32] Grippaudo C, Garcovich D, Volpe G, Lajolo C (2006) Comparative evaluation between cervical vertebral morphology and hand-wrist morphology for skeletal maturation assessment. Minerva Stomatol 55:16688103

[33] Cericato GO, Bittencourt MAV, Paranhos LR (2015). Validity of the assessment method of skeletal maturation by cervical vertebrae: a systematic review and meta-analysis. Dentomaxillofacial Radiol.

[34] Cardoso HFV, Saunders SR (2008). Two arch criteria of the ilium for sex determination of immature skeletal remains: a test of their accuracy and an assessment of intra- and inter-observer error. Forensic Sci Int.

[35] Suazo Galdames IC, Zavando Matamala DA, Smith RL (2008). Blind test of mandibular morphology with sex indicator in subadult mandibles. Int J Morphol.

[36] Blake KAS (2019). A test of sex estimation in subadults using the elevation of the auricular surface from four samples of known age and sex. J Forensic Sci.

